# ISGylation controls exosome secretion by promoting lysosomal degradation of MVB proteins

**DOI:** 10.1038/ncomms13588

**Published:** 2016-11-24

**Authors:** Carolina Villarroya-Beltri, Francesc Baixauli, María Mittelbrunn, Irene Fernández-Delgado, Daniel Torralba, Olga Moreno-Gonzalo, Sara Baldanta, Carlos Enrich, Susana Guerra, Francisco Sánchez-Madrid

**Affiliations:** 1 Centro Nacional de Investigaciones Cardiovasculares (CNIC), 28029 Madrid, Spain; 2 Immunology Service, Hospital de la Princesa, Instituto Investigación Sanitaria Princesa, Universidad Autónoma de Madrid, 28006 Madrid, Spain; 3 Department of Preventive Medicine, Public Health and Microbiology, Universidad Autónoma de Madrid, 28029 Madrid, Spain; 4 Departament de Biomedicina, Unitat de Biologia Cel·lular, Centre de Recerca Biomèdica CELLEX, Institut d'Investigacions Biomèdiques August Pi i Sunyer (IDIBAPS), Facultat de Medicina, Universitat de Barcelona, 08036 Barcelona, Spain

## Abstract

Exosomes are vesicles secreted to the extracellular environment through fusion with the plasma membrane of specific endosomes called multivesicular bodies (MVB) and mediate cell-to-cell communication in many biological processes. Posttranslational modifications are involved in the sorting of specific proteins into exosomes. Here we identify ISGylation as a ubiquitin-like modification that controls exosome release. ISGylation induction decreases MVB numbers and impairs exosome secretion. Using ISG15-knockout mice and mice expressing the enzymatically inactive form of the de-ISGylase USP18, we demonstrate *in vitro* and *in vivo* that ISG15 conjugation regulates exosome secretion. ISG15 conjugation triggers MVB co-localization with lysosomes and promotes the aggregation and degradation of MVB proteins. Accordingly, inhibition of lysosomal function or autophagy restores exosome secretion. Specifically, ISGylation of the MVB protein TSG101 induces its aggregation and degradation, being sufficient to impair exosome secretion. These results identify ISGylation as a novel ubiquitin-like modifier in the control of exosome production.

## Abstract (web summary)

Multivesicular bodies (MVB) are endosomal compartments that can either fuse with the plasma membrane for the secretion of exosomes, or fuse with the lysosome and be degraded along with their contents. Here, the authors show that ISGylation of the MVB protein TSG101 impairs exosome secretion and acts as a regulator of MVB fate.

## Graphical abstract

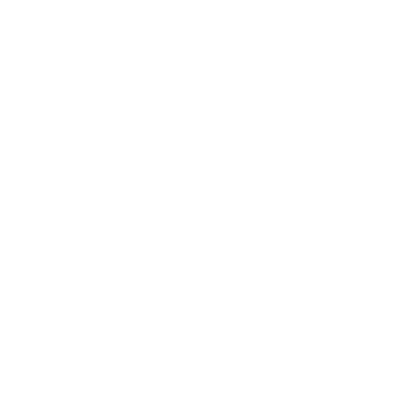

Exosomes are vesicles secreted to the extracellular environment by most cell types. They are key mediators of cell-to-cell communication in many different contexts, including the immune response^1^,^2^ and tumour progression^3^,^4^. Exosomes originate in endosomal compartments called multivesicular bodies (MVBs), which are late endosomes containing multiple intraluminal vesicles (ILVs) formed by the invagination of the endosomal membrane. When MVBs fuse with the plasma membrane, ILVs are released as exosomes^5^. Alternatively, MVBs can fuse with the lysosomal compartment, resulting in degradation of their content.

Exosome composition is not a mere copy of cytosolic content; rather, specific proteins and nucleic acids are selectively sorted into exosomes. The amount and content of exosomes can moreover change in response to different stimuli^6^,^7^. Such changes in exosome composition determine the final outcome of exosome-mediated communication^8^,^9^.

The mechanisms that control exosome composition and content are still not well understood^10^. Posttranslational modifications such as ubiquitination may play an important role in the sorting of proteins into exosomes^11^,^12^,^13^. The endosomal sorting complex required for transport (ESCRT) recognizes ubiquitinated proteins and sorts them into ILVs^14^. The ESCRT complex is essential for the sorting of proteins such as epidermal growth factor receptor into MVBs that are degraded through fusion with lysosomes^15^, but is also involved in the regulation of exosome composition and secretion^16^,^17^. Another ubiquitin-like protein (UBL) that can modify exosomal proteins is SUMO, whose conjugation to hnRNPA2B1 is essential for the sorting of microRNAs into exosomes^18^, and enhances the secretion of α-synuclein into extracellular vesicles (EVs) in an ESCRT-dependent manner^19^.

ISG15 is an interferon (IFN)-α/β-induced UBL^20^, which exerts its functions in two distinct states: as a free molecule (intracellular and extracellular)^21^ or conjugated to target proteins (ISGylation)^22^,^23^. Analogous to ubiquitin, ISG15 conjugation is mediated by the consecutive action of an E1-activating enzyme (Ube1L), an E2-conjugating enzyme (UbCH8) and E3 ligases (mHERC6/hHERC5)^24^,^25^,^26^, and counteracted by the specific isopeptidase USP18 (ref. 27). ISGylation was shown to occur in a co-translational process favouring modification of viral proteins in infected cells, which, in turn, interferes with virus assembly or function^28^,^29^,^30^. Furthermore, cellular proteins involved in antiviral defense or export of viral particles have been shown to be ISGylated, supporting the antiviral function of ISG15 (ref. 28). Studies in mice have demonstrated a role for ISG15 in antiviral immunity. Hence, mice lacking ISG15 exhibit a higher susceptibility to several pathogens including virus^31^ and bacteria^32^, and this is reverted in USP18-mutant mice, in which high levels of ISG15 conjugation are observed^33^. However, human ISG15 seems to have critical immune functions but not in antiviral immunity; unlike mice, ISG15 deficiency increased viral resistance in humans^34^. Specifically, free extracellular human ISG15 is important in IFN-γ-dependent anti-mycobacterial immunity^21^, whereas free intracellular ISG15 is involved in USP18-mediated downregulation of IFN-α/β signalling^35^.

ISG15 expression blocks the process of virus-budding by different mechanisms such as the blockage of ESCRT machinery in HIV-infected cells^36^, or in the case of Ebola and other enveloped virus infections, inhibiting the Nedd4 E3 ubiquitin ligase^37^. Interestingly, exosomes and viruses share many features, and some viruses have been shown to exploit exosome and microvesicle secretion pathways^38^,^39^. In addition, exosomes are enriched in ISGylation targets, such as TSG101 (ref. 40) and heat-shock proteins^41^.

Here we show that IFN-I inhibits exosome secretion by inducing protein ISGylation. We demonstrate that the ISGylation of the MVB protein TSG101 induces its aggregation and degradation, and this is sufficient for impairing exosome secretion. Moreover, the ISGylation-induced defect in exosome secretion is rescued on inhibition of lysosomal function or autophagy.

## Results

### ISGylation inhibits exosome secretion

**Figure 1:**
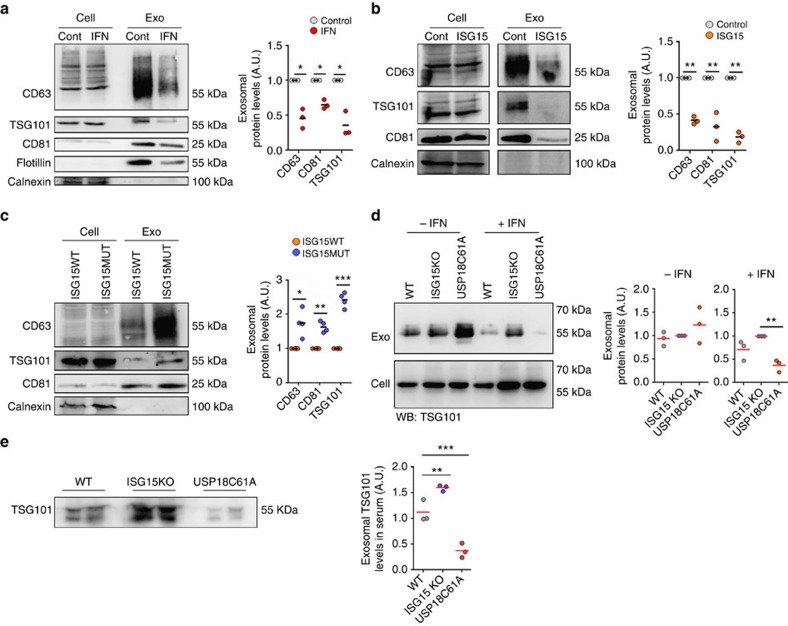
ISGylation inhibits exosome secretion. (**a**) Western blot analysis of EVs purified by serial ultracentrifugation from cell culture supernatants from equal numbers of Jurkat T cells untreated (Cont) or treated with 1,000 U ml^−1^ IFN-I for 16 h. Cells and EVs (Exo) were blotted for the exosomal markers CD63, TSG101, Flotillin and CD81, and for the endoplasmic reticulum marker Calnexin. Right graph: quantification of exosomal protein levels in the EVs obtained from IFN-I-treated and untreated cells in three independent experiments. (**b**) Western blot analysis of the EVs obtained from equal numbers of untransfected HEK293 cells (Cont) or co-transfected with ISG15 and the ISGylation machinery; E1, E2, E3 ligases (ISG15). Cells and EVs (Exo) were blotted for CD63, TSG101, CD81 and Calnexin. Right graph: quantification of exosomal protein levels in the EVs obtained from untransfected HEK293 cells or co-transfected with ISG15 and the ISGylation machinery in three independent experiments. (**c**) Western blot analysis of the EVs obtained from equal numbers of HEK293 cells co-transfected with plasmids encoding the ISGylation machinery and the functional (ISG15WT) or mutated ISG15 (ISG15MUT). Cells and EVs (Exo) were blotted for CD63, CD81 and Calnexin. Right graph: quantification of exosomal protein levels in four independent experiments. (**d**) Western blot analysis of the EVs obtained from equal numbers of *WT*, *ISG15KO* and *USP18C61A* BMDMs treated 16 h with IFN-I or left untreated. Cells and EVs were blotted for TSG101 and quantification of exosomal protein levels of IFN-I-treated and -untreated cells is shown for three independent experiments. (**e**) Western blot analysis of the EVs obtained in blood serum from poly(I:C)-injected *WT*, *ISG15KO* and *USP18C61A* mice. EVs were isolated from 250 μl of serum and blotted for TSG101. Right graphs: quantification of exosomal TSG101 protein levels of three mice per genotype; *t*-test *P-value<0.05, **P-value<0.001 and ***P-value<0.0001.

To analyse the role of ISGylation on exosome production, we first treated Jurkat T cells with IFN-I, to induce ISG15 expression and conjugation (Supplementary Fig. 1A), and purified the secreted EVs from their culture supernatants by a serial ultracentrifugation protocol. IFN-I treatment inhibited the secretion of the classical exosome markers CD63, TSG101 and CD81 in the purified EVs (Fig. 1a). A concomitant decrease in the exosomal markers on IFN-I treatment was also observed after an additional purification step by ultracentrifugation in a sucrose gradient (Supplementary Fig. 1B). Interestingly, the few EVs secreted by IFN-treated cells were recovered in different fractions than the EVs from non-treated cells, suggesting that they might be of different nature. To further investigate the function of ISGylation in controlling EVs secretion, we next overexpressed ISG15 and the machinery responsible for ISGylation (E1, E2 and E3) in HEK293 cells, which mimics the activation of the ISG15 pathway (Supplementary Fig. 1A)^24^,^28^ without activating other pathways induced by IFN-I treatment. ISGylation induction inhibited the secretion of EVs containing CD63, TSG101 and CD81 (Fig. 1b). To dissect whether the inhibition in exosomal markers secretion was due to ISG15 overexpression itself or to increased ISG15 conjugation to proteins, we overexpressed the ISGylation machinery together with an ISG15 mutated at the carboxy-terminal (ISG15MUT), thus disabling protein conjugation. Cells overexpressing ISG15MUT showed higher levels of exosomal markers in comparison with ISG15WT (Fig. 1c), demonstrating that ISG15 conjugation to proteins, but not free ISG15, is required for the inhibition of exosomal markers secretion. Nanoparticle tracking analysis (NTA) showed a decrease in the number of particles secreted on ISG15WT overexpression in comparison with ISG15MUT-expressing cells (Supplementary Fig. 1C), indicating that ISGylation is not causing a mere decrease in the sorting of exosomal markers in EVs, but an actual decrease in exosome secretion. However, NTA did not reveal any significant decrease in the secretion of nanoparticles on IFN-I treatment (Supplementary Fig. 1D).

To study the role of ISG15 in exosome secretion in a more physiological context, we analysed the secretion of exosomes by primary cells from wild-type (*WT*), ISG15 knockout (*ISG15KO*)^42^ and *USP18C61A* mice, which have the ISG15 de-conjugating enzyme USP18 enzymatically inactive and thus show higher levels of protein ISGylation^33^. Consistent with the inhibition of exosome secretion induced by ISGylation, IFN-I treatment decreased exosome secretion in primary bone marrow-derived macrophages (BMDMs) from *WT* mice but not in ISG15-deficient macrophages (Fig. 1d). Furthermore, BMDMs from *USP18C61A* mice secrete less exosomes than either *WT* or *ISG15KO* mice on ISGylation induction by IFN-I, supporting the role of ISG15 protein conjugation in the regulation of exosome secretion. Similar results were also obtained with primary T lymphoblasts (Supplementary Fig. 1E). To assess the role of ISGylation *in vivo,* poly (I:C) was injected intraperitoneally into *WT*, *ISG15KO* and *USP18C61A* mice, to induce ISG15 expression^43^, and exosomes from blood serum analysed. Interestingly, there were less exosomes in serum from poly (I:C)-injected *WT* than *ISG15KO* mice, and even less exosomes in serum from injected *USP18C61A* mice (Fig. 1e), demonstrating the role of ISGylation in the inhibition of exosome secretion *in vivo*. Overall, our gain- and loss-of-function experiments strongly support the role of ISGylation in the regulation of exosome secretion *in vitro* and *in vivo*.

### ISGylation decreases MVB numbers

**Figure 2:**
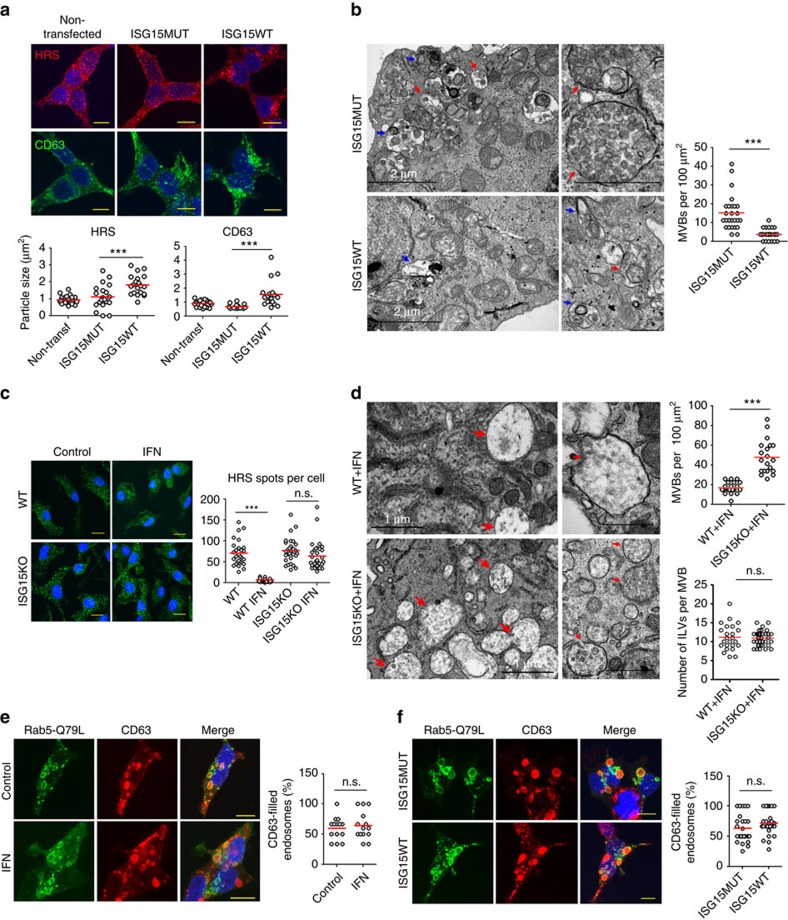
ISGylation decreases MVB numbers. (**a**) Confocal microscopy analysis of the endosome markers HRS (red) and CD63 (green) in non-transfected HEK293 cells or HEK293 cells co-transfected with plasmids encoding the ISGylation machinery and the functional (ISG15WT) or mutated ISG15 (ISG15MUT). Scale bar, 10 μm. Right graph: quantification of HRS^+^ and CD63^+^ average particle size per cell (*n*=20). Each dot represents the average particle size from individual cells and mean is indicated in red lines. (**b**) Electron microscopy images showing representative fields with MVBs (red arrows) and lysosomes/autophagosomes (blue arrows) in HEK293 transfected as in **a**. Right graph: quantification of MVB numbers in more than 25 fields per condition. Each dot represents the number of MVBs per section and mean is indicated in red lines. Scale bar (insets), 500 nm. (**c**) Confocal microscopy analysis of HRS in *WT* or *ISG15KO* BMDMs left untreated or treated with 1,000 U ml^−1^ IFN for 16 h. Scale bar, 10 μm. Right graph: quantification of the number of HRS^+^ particles per cell. Each dot represents the number of HRS^+^ particles from individual cells and mean is indicated in red lines (*n*=28). (**d**) Electron microscopy images showing MVBs (red arrows) in *WT* or *ISG15KO* BMDMs treated with 1,000 U ml^−1^ IFN for 16 h. Upper graph, quantification of MVB numbers in 20 fields per condition. Each dot represents the number of MVBs per section and mean is indicated in red lines. Lower graph, quantification of ILV numbers per MVB. Each dot represents the number of ILVs per MVB and mean is indicated in red lines (*n*≥24). Scale bar (insets), 500 nm. (**e**) Confocal microscopy analysis of CD63 (red) in Rab5-Q79L-GFP^+^ endosomes (green). HEK293 cells were transfected with Rab5-Q79L-GFP and treated 16 h with 1,000 U ml^−1^ IFN or left untreated. (**f**) Confocal microscopy analysis of CD63 (red) in Rab5-Q79L-GFP^+^ endosomes (green). HEK293 cells were co-transfected with Rab5-Q79L-GFP mutant, the ISGylation machinery and ISG15WT or ISG15MUT. Images from **e**,**f**: Scale bar, 10 μm. Right graphs: each dot represents the percentage of CD63-filled endosomes per individual cell and mean is indicated in red lines; *t*-test; NS: *P*-value>0.05 and ***P-value<0.0001.

To investigate the mechanisms by which ISGylation controls exosome secretion, MVBs were studied. Hepatocyte growth factor-regulated tyrosine kinase substrate (HRS) is involved in MVB formation and exosome secretion^17^,^44^, and it has been previously used as a marker of MVBs, being present in intermediates between early and late MVBs positive for TSG101 and LBPA^44^,^45^. ISGylation induction resulted in a more perinuclear and clustered localization of HRS, as shown by the increase in the size of HRS^+^ structures (Fig. 2a), and decreased the number of HRS^+^ spots per cell (Supplementary Fig. 2A). Similar results were obtained with the MVB marker CD63 (Fig. 2a and Supplementary Fig. 2A), whereas the early endosome (EE) marker EEA1 did not show any apparent alteration (Supplementary Fig. 2B). Electron microscopy analyses revealed significant reduced MVBs numbers and density on ISGylation induction (Fig. 2b). In accordance, IFN-I treatment reduced HRS^+^ spots in WT macrophages but not in *ISG15KO* macrophages (Fig. 2c), and MVBs were more abundant in *ISG15KO* macrophages than in their WT counterparts on IFN-I treatment (Fig. 2d), overall suggesting a role for ISG15 in regulating MVB numbers.

To ascertain whether ISGylation decreases MVB numbers by affecting MVB formation, we transfected HEK293 cells with the constitutively active Rab5-Q79L-GFP mutant, which forms large endosomes with a mixed morphology between EE and MVBs, which facilitates the study of the first steps in MVB biogenesis^46^,^47^. ISGylation induction by either IFN-I or ISG15 overexpression did not affect the sorting of the exosomal protein CD63 into Rab5-Q79L-GFP endosomes (Fig. 2e,f). Furthermore, quantitative electron microscopy analyses did not show any significant differences in the number of ILVs per MVB on ISGylation induction (Supplementary Fig. 2C,D). To rule out any possible side effect derived from the uneven distribution of small ILVs within enlarged MVBs^48^, ILV numbers were also quantified in the absence of Rab5-Q79L-GFP overexpression. Accordingly, macrophages from *WT* and *ISG15KO* mice also showed similar levels of ILVs per MVB on ISGylation induction by IFN (Fig. 2d). Altogether, these data support that ISGylation does not impair the formation of ILVs, suggesting that it is mainly affecting later stages on the MVB pathway.

### ISGylation induces protein aggregation and degradation

We then sought to understand how ISGylation regulates MVB numbers and exosome secretion. For this, we used ISG15 fusion proteins as models of protein ISGylation, as previously described for other posttranslational modifications such as ubiquitin or other UBLs^49^,^50^,^51^. Although fusion proteins are not exactly the same than endogenous ISGylation in lysines, in our ISG15 fusion proteins the C-terminal glycine of ISG15 is fused to the NH_2_ group of the target protein in the N-terminal amino acid through a peptidic bond, which is chemically analogue to the isopeptidic bond formed between the endogenous ISG15 and the NH_2_ group of a target protein lysine.

**Figure 3:**
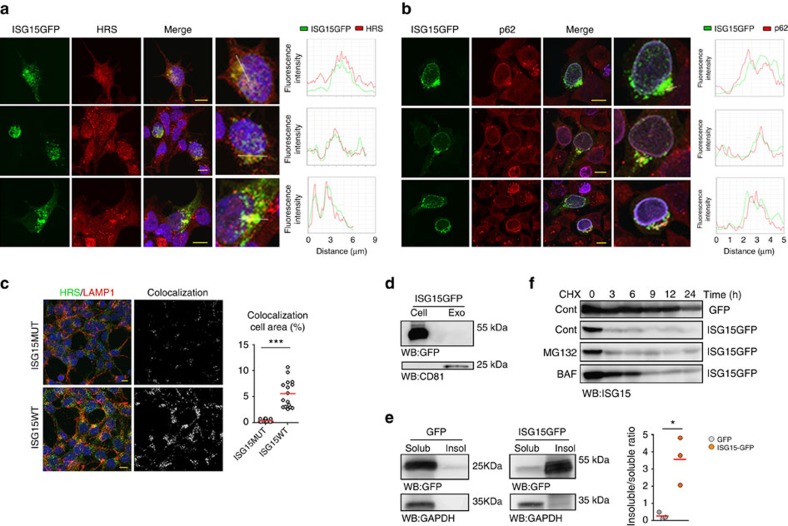
ISG15 conjugation induces protein aggregation and degradation by lysosomes. (**a**) Confocal co-localization analysis of ISG15-GFP (green) and the MVB marker HRS (red). Right graphs: fluorescence intensity profiles of ISG15-GFP (green) and HRS (red) in the regions delineated by a white line. Nuclei were stained with DAPI. Scale bar, 10 μm. (**b**) Confocal co-localization analysis of ISG15-GFP (green) and p62 (red). Right graphs represent fluorescence intensity profiles of ISG15-GFP (green) and p62 (red) of the regions delineated by a white line. Nuclei were stained with DAPI. Scale bar, 10 μm. (**c**) Confocal microscopy analysis of HRS (green) and LAMP1 (red) co-localization in HEK293 cells co-transfected with ISGylation machinery and functional (ISG15WT) or mutated ISG15 (ISG15MUT). Nuclei were stained with DAPI. Scale bar, 10 μm. Co-localization area per cell was quantified by ImageJ (*n*=16). Each dot represents the percentage of co-localization area per cell surface and mean is indicated in red lines. (**d**) Western blot analysis of exogenous ISG15 in HEK293 cells transfected with ISG15-GFP. Cells and EVs (EXO) were blotted for GFP and CD81. (**e**) Western blot analysis of ISG15-GFP and GFP in 0.5% NP-40 soluble and insoluble cell fractions in HEK293 transfected with GFP or ISG15-GFP. Right graph: quantification of GFP and ISG15-GFP in the insoluble fraction respect to the soluble fraction in three independent experiments. (**f**) Western blot analysis of GFP and ISG15-GFP degradation kinetics in HEK293 cells transfected with GFP or ISG15-GFP and treated with cycloheximide to inhibit protein synthesis during the indicated times. Where specified, the medium was supplemented with the lysosome inhibitor Bafilomycin A1 (BAF) or the proteasome inhibitor MG132. A representative blot from two independent experiments is shown. Data from **c**,**e**: *t*-test *P-value<0.05 and ***P-value<0.0001.

Confocal analysis showed partial co-localization of ISG15-GFP fusion protein with the MVB marker HRS and with the aggresome marker p62 (ref. 52 and Fig. 3a,b), whereas no co-localization was detected with the EE marker EEA1 (Supplementary Fig. 3A). Notably, ISGylation increased the co-localization of HRS with the lysosome marker LAMP1 (Fig. 3c), although neither LAMP1 content nor localization were affected (Supplementary Fig. 3B).

Despite its co-localization with MVB markers, green fluorescent protein (GFP) was not found in exosomes when fused to ISG15 (Fig. 3d) but rather promoted its accumulation in detergent-insoluble cell fractions (Fig. 3e), indicating that ISGylation promotes protein aggregation. Western blot analysis of cycloheximide-treated cells showed that when fused to ISG15, GFP is degraded faster than GFP alone or when fused to the ubiquitin-like modifier SUMO2, and that this degradation is delayed by the lysosome inhibitor Bafilomycin A1 but not by the proteasome inhibitor MG132 (Fig. 3f and Supplementary Fig. 3C–E), thus indicating that ISGylation of proteins promotes their degradation through the lysosome.

### Inhibition of MVB–lysosome fusion rescues exosome secretion

**Figure 4:**
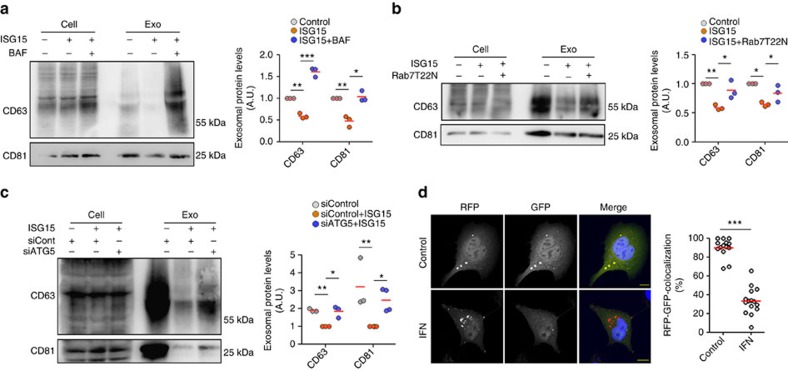
Inhibition of lysosome function upon ISGylation induction rescues exosome secretion. (**a**) Western blot analysis of exosome secretion in untransfected HEK293 cells and HEK293 cells co-transfected with plasmids encoding the ISGylation machinery and ISG15, and treated with Bafilomycin A1 (BAF), when indicated. Cells and EVs (Exo) were blotted for CD63 and CD81. Right graph: quantification of exosomal protein levels in three independent experiments. (**b**) Western blot analysis of exosome secretion in HEK293 cells transfected with GFP or co-transfected with plasmids encoding ISG15 and the ISGylation machinery, and when indicated, with the Rab7T22N dominant-negative mutant. Cells and EVs were blotted for CD63 and CD81. Right graph: quantification of exosomal protein levels in three independent experiments. (**c**) Western blot analysis of exosome secretion in HEK293 cells transfected with control small interfering RNAs (siRNAs) or siRNAs targeting ATG5. After 48 h, cells were re-transfected with siRNAs together with plasmids encoding ISG15 and ISGylation machinery. Cells and EVs (Exo) were blotted for CD63 and CD81. Right: quantification of exosomal protein levels in three or four independent experiments. (**d**) Confocal analysis of LC3 localization into acidic compartments in HEK293 cells transfected with LC3-RFP-GFP tandem plasmid and treated 16 h with IFN-I where indicated. Nuclei were stained with DAPI. Scale bar, 10 μm. Images were processed by ImageJ and the number of RFP^+^ and RFP^+^GFP^+^ dots quantified. Each dot represents the percentage of RFP^+^GFP^+^ dots per individual cell and mean is indicated in red lines; *t*-test; ***P-value<0.001. Data **a**,**b**,**c**: analysis of variance; *P-value<0.05, **P-value<0.001 and ***P-value<0.0001.

To assess whether ISGylation inhibits exosome secretion by inducing MVB lysosomal degradation, we studied whether inhibition of MVB fusion with lysosomes could recover exosome secretion. Bafilomycin A1 is a proton pump inhibitor that increases the pH of lysosomes and inhibits trafficking between MVB and lysosomes^53^,^54^. Bafilomycin A1 treatment recovered exosome secretion in HEK293 cells in which ISGylation was induced by ISG15 overexpression (Fig. 4a), whereas it did not increase exosome secretion in control cells (Supplementary Fig. 4A), suggesting that ISGylation inhibits exosome secretion by re-routing MVB to lysosomes but is not impairing MVB biogenesis. This is also supported by the detection of ISGylated proteins in exosomes on secretion recovery (Supplementary Fig. 4B). Exosome secretion was also recovered despite ISGylation induction by IFN-I in Jurkat T cells when treated with Bafilomycin A1 (Supplementary Fig. 4C).

To avoid unspecific effects derived from the use of the inhibitor, we also studied exosome recovery on Rab7T22N dominant-negative mutant expression, which inhibits both endosome–lysosome and autophagosome–lysosome fusion^55^,^56^,^57^,^58^. Rab7T22N expression rescued exosome secretion in HEK293 cells despite ISGylation induction by ISG15 overexpression (Fig. 4b), whereas it did not increase exosome secretion in control cells (Supplementary Fig. 4D), supporting that the ISGylation-induced inhibition of exosome secretion is mediated by MVB fusion and degradation by the lysosome.

Fusion of MVBs with autophagosomes on induction of autophagy by rapamycin has been reported^56^. Interestingly, the secretion of exosomes on ISGylation induction was also recovered on silencing of the autophagy mediator ATG5 (Fig. 4c), indicating that autophagy probably contributes to the decreased exosome release induced by ISGylation. Accordingly, IFN-I treatment promoted the localization of the autophagy mediator LC3 in more acidic compartments, presumably lysosomes, supported by the loss of GFP fluorescent signal in mRFP-GFP-LC3 tandem reporter (Fig. 4d). Notably, ISGylation induction by IFN-I treatment or ISG15 overexpression did not induce general autophagy, as evidenced by unaltered levels of p62 and other autophagy markers (Supplementary Fig. 4E,F), indicating that ISGylation promotes selective autophagy and degradation of MVB without inducing a global autophagy response.

### ISG15 impairs exosome secretion by modifying TSG101

**Figure 5:**
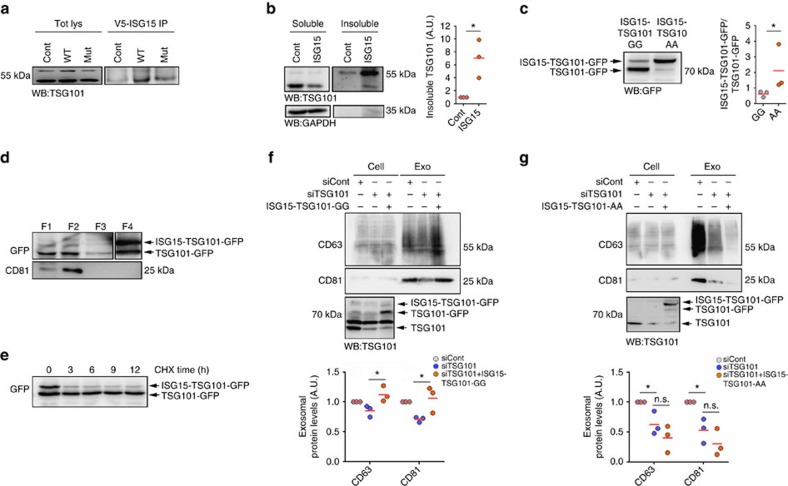
ISG15 impairs exosome secretion by modifying TSG101. (**a**) Western blot analysis of TSG101 immunoprecipitation in HEK293 cells non-transfected or HEK293 cells co-transfected with plasmids encoding the ISGylation machinery and functional (ISG15WT) or mutated ISG15 (ISG15MUT). Total lysates and V5 immunoprecipitates were blotted for TSG101. (**b**) Western blot analysis of 0.5% NP-40 soluble and insoluble fractions from non-transfected HEK293 cells (Cont) or cells transfected with ISG15 and ISGylation machinery (ISG15). Soluble and insoluble fractions were blotted for TSG101 and GAPDH. Right graph: quantification of TSG101 protein levels in insoluble fractions in three independent experiments. (**c**) Western blot analysis of TSG101-GFP ISGylation in HEK293 cells transfected with ISG15-TSG101-GFP-GG or with the non-de-ISGylable mutant ISG15-TSG101-GFP-AA. Cell lysates were blotted for GFP. Right graph: quantification of ISG15-TSG101-GFP/TSG101-GFP ratio in three independent experiments. (**d**) Western blot analysis of ISG15-TSG101-GFP and TSG101-GFP subcellular distribution in HEK293 cells transfected with ISG15-TSG101-GFP-GG. Fractions corresponding to cytosol (F1), membranes (F2), nucleus (F3) and insoluble aggregates (F4) were extracted and blotted for GFP and CD81. A lower exposition of the same blot is shown for F4. (**e**) Western blot analysis of ISG15-TSG101-GFP and TSG101-GFP degradation kinetics in HEK293 cells transfected with ISG15-TSG101-GFP-GG and treated with cycloheximide during the indicated times. Cell lysates are blotted for GFP. (**f**) Western blot analysis of HEK293 cells transfected with control small interfering RNAs (siRNAs) or siRNAs targeting TSG101. When indicated, cells were co-transfected with ISG15-TSG101-GFP-GG. Cells and EVs (EXO) were blotted for CD63 and CD81. Lower gel shows cell lysates blotted for TSG101 to check TSG101 silencing and exogenous overexpression. Lower graph: quantification of exosomal protein levels in three independent experiments. (**g**) Western blot analysis of HEK293 cells transfected with control siRNAs or siRNAs targeting TSG101. When indicated, cells were co-transfected with the non-de-ISGylable mutant ISG15-TSG101-GFP-AA. Cells and EVs (EXO) were blotted for CD63 and CD81. Lower gel shows cell lysates blotted for TSG101 to check TSG101 silencing and exogenous overexpression. Lower graph: quantification of exosomal protein levels in three independent experiments. Data **b**,**c**: *t*-test; NS: *P*-value>0.05 and *P-value<0.05. Data **f**,**g**: analysis of variance; NS: *P*-value>0.05 and *P-value<0.05.

To elucidate the molecular mechanism by which ISGylation regulates exosome secretion, we focused on TSG101, a component of the ESCRT machinery involved in exosome biogenesis that has been shown to be ISGylated and to regulate the secretion of virus^40^. To confirm TSG101 ISGylation, we overexpressed the ISGylation machinery together with *WT* or mutated ISG15 fused to the V5 tag and performed V5 immunoprecipitation. Western blot analysis of TSG101 in V5-ISG15 but not in control or V5-ISG15MUT immunoprecipitates confirmed endogenous TSG101 ISGylation (Fig. 5a). Moreover, ISGylation induction by ISG15 overexpression induced endogenous TSG101 aggregation and accumulation in insoluble fractions (Fig. 5b). To address whether ISGylation of TSG101 is sufficient to inhibit exosome secretion, we generated a TSG101-GFP plasmid that mimics TSG101 ISGylation (referred as ISG15-TSG101-GG), in which ISG15 terminal glycine is conjugated to TSG101 N-terminal through a peptide bound that can be cleaved by the USP18 de-ISGylase (Supplementary Fig. 5A). Consequently, both ISGylated and de-ISGylated TSG101-GFP are detected in cells transfected with this plasmid (Fig. 5c). Interestingly, ISGylated TSG101-GFP accumulated in insoluble fractions and was degraded faster than de-ISGylated TSG101-GFP (Fig. 5d,e), consistent with ISGylation promoting TSG101 protein aggregation and degradation. The expression of the ISG15-TSG101-GG plasmid rescued exosome secretion in cells targeted for endogenous TSG101 (Fig. 5f and Supplementary Fig. 5B), probably due to the presence of a functional de-ISGylated form of TSG101. To circumvent this, we generated a non-de-ISGylable TSG101 mutant by changing ISG15 terminal glycines into alanines (ISG15-TSG101-AA), thus preventing TSG101 de-ISGylation (Fig. 5c). In cells devoid of endogenous TSG101, overexpression of the non-de-ISGylable TSG101 mutant did not restore the secretion of exosomes (Fig. 5g and Supplementary Fig. 5C), supporting that ISG15 conjugation to the ESCRT component TSG101 is sufficient to inhibit the secretion of exosomes.

## Discussion

Cells secrete a variety of EVs including shedding vesicles, coming from the direct evagination of the plasma membrane, and exosomes, coming from endosomal compartments called MVBs. The membrane of MVB invaginates to form ILVs, which are secreted to the extracellular environment on MVB fusion with the plasma membrane^5^. MVBs can also fuse with the lysosomal compartment for the degradation of their content. There is evidence that different types of MVB co-exist in cells^59^, although the differences in their composition and the mechanisms that control their biogenesis and final fusion with the lysosome or plasma membrane are not well understood^10^. In addition, on specific stimuli such as starvation or rapamycin treatment, MVBs can be re-routed to promote their fusion with the lysosomal compartment and thus avoid their fusion with the plasma membrane and the concomitant secretion of exosomes^56^ demonstrating that the final fate of MVBs is not immutable but can change under specific conditions^60^.

The role of the ESCRT complex in the sorting of ubiquitinated proteins into ILVs for their degradation in the lysosome is well known^61^ and some of the ESCRT complex components are also involved in the secretion of exosomes and other EVs^12^,^17^, although their precise role in the biogenesis of the different types of EVs is not known. Other ubiquitin-like modifiers have been shown to control the composition of EVs, for example, SUMOylation of hnRNPA2B1 promotes the sorting of specific microRNAs into exosomes^18^, and also controls the sorting of α-synuclein into EVs^19^. Here we identify the ubiquitin-like modifier ISG15 as a novel regulator of the exosome pathway. We show that ISGylation induction by ISG15 machinery overexpression or IFN-I decreases the secretion of exosomal markers. Importantly, experiments with the non-conjugable ISG15MUT and with primary cells from mice with defective function of the specific ISG15 de-ISGylase USP18 demonstrated that this effect is mediated by ISG15 conjugation to proteins and not by free ISG15. ISGylation induction by ISG15 overexpression induces a decrease in microparticle secretion, indicating that ISGylation is not merely decreasing the sorting of certain exosomal markers into ILVs but is actually decreasing exosome secretion. However, ISGylation induction by IFN-I does not induce a significant decrease in the number of secreted microparticles. This could be due to the induction of the secretion of other types of EVs by IFN-I and this is in agreement with previous reports showing that IFN-I can promote the secretion of EVs loaded with antiviral components that are transferred to other cells^62^. In fact, on ultracentrifugation in sucrose gradients, the few EVs secreted by IFN-treated cells are recovered in different fractions than the EVs from non-treated cells, suggesting that they might have a different nature. Alternatively, the absence of effect of IFN on global particle secretion may indicate that only the EVs derived from MVBs are affected.

We also show that ISGylation decreases the numbers of MVBs, but does not prevent the formation of ILVs. The secretion of exosomes is recovered when the fusion of MVB with lysosomes or autophagosomes is inhibited, indicating that the observed inhibition of exosome secretion is mainly mediated by the induction of MVB degradation by the lysosome. In accordance, we show that ISGylation promotes the aggregation and degradation of proteins by the lysosome, and this is in agreement with previous reports in which ISG15-linked proteins associated with the proteins p62 and HDAC-6 involved in the autophagic process^52^. We have observed that ISGylation decreases the number of HRS^+^ structures without preventing the formation of ILVs, possibly reflecting an accelerated degradation of endosomes. In addition, ISGylation increases the co-localization of the lysosome marker LAMP-1 with HRS, which is usually absent from LAMP-1-positive structures^44^. Whether ISGylation of MVB proteins induces the rapid fusion of MVB with lysosomes in an earlier stage of maturation, or whether it directly impairs HRS dissociation from endosomal membranes is not known.

**Figure 6:**
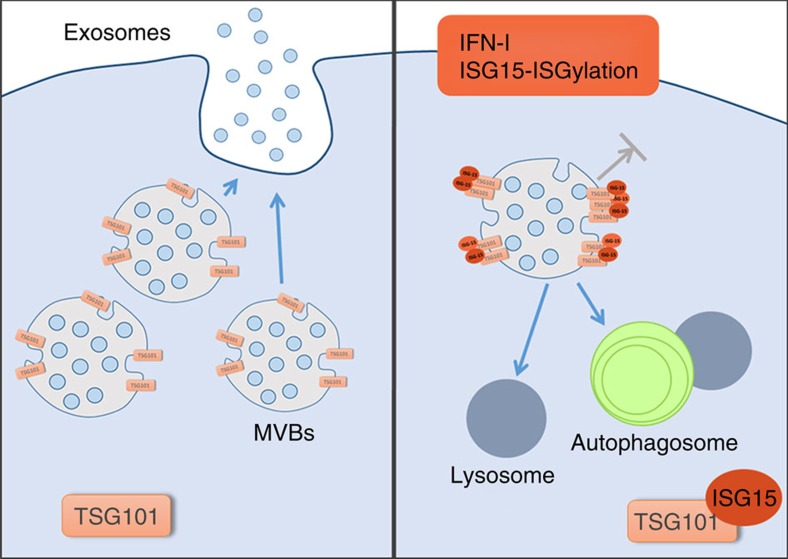
Proposed model for the role of ISGylation in the regulation of exosome secretion. IFN-I induces ISG15 expression and conjugation to MVB proteins such as TSG101. ISGylation of MVB proteins promotes MVB fusion with lysosomes and degradation, thus inhibiting exosome secretion.

A number of ISG15 target proteins are typically present in exosomes and MVBs^41^, including several components of the ESCRT complex such as TSG101, CHMP2A, CHMP4B and CHMP6 (refs 40, 63, 64). It is therefore conceivable that ISG15 is recruited to MVB by conjugating endosomal proteins. There, it may promote protein aggregation and enhance MVB degradation by the autophagosome–lysosome compartment, thus preventing their fusion with the plasma membrane and the secretion of exosomes (Fig. 6). We have validated the ISGylation of TSG101, which has been previously shown to control the secretion of viral particles^40^, and we show that TSG101 ISGylation is sufficient to impair exosome secretion. However, we cannot rule out the possibility that other endosomal proteins are being ISGylated and also contribute to the inhibition of exosome secretion induced by ISG15. TSG101 depletion has been previously shown to inhibit MVB formation^65^ or to modify the size of MVBs and/or the number of ILVs^48^. However, there are significant differences between depletion and ISGylation of TSG101. ISG15 may be directed to endosomes in a later stage of their maturation and here modify MVB proteins such as TSG101, and promote their aggregation and subsequent degradation by the lysosome without impairing ILV biogenesis. However, we could not completely rule out any effect of ISGylation on MVB biogenesis.

ISGylation has been previously shown to exert an antiviral activity by blocking the exit of viral particles^36^,^40^,^64^,^66^. The ISGylation of multiple MVB proteins and the consequent inhibition of exosome secretion induced by IFN-I would prevent the spreading of viruses that exploit the MVB pathway for their way out the cell. In addition, exosomes play important roles in cell-to-cell communication during the immune response, tumour progression, neuron survival and many other contexts^1^,^3^,^4^,^67^. Therefore, ISGylation induction by different stimuli such as viral infection, IFN, ischaemia^68^ or ageing^69^ can be an important mechanism to regulate exosome-mediated communication in many different situations.

## Methods

### Cell culture

The human Jurkat-derived T-cell line J77cl20 (TCR Vαl. 2 Vβ8) was cultured in RPMI (Sigma) containing 10% fetal bovine serum (FBS; Invitrogen). The HEK293T cell line was cultured in DMEM medium (Sigma) containing 10% FBS (Invitrogen).

Mouse primary macrophages (BMDMs) were obtained from the bone marrow of C57BL/6 *WT*, ISG15 knockout^42^ and *USP18C61A* mice^33^ (kindly provided by K. Knobeloch, Freiburg University, Germany). Cells were cultured RPMI-1640 supplemented with macrophage colony-stimulating factor (30% mycoplasma-free L929 cell supernatant, NCBI Biosample accession number SAMN00155972) for 6 days, to induce cell differentiation.

Mouse primary T lymphocytes were obtained from cell suspensions prepared from spleens and peripheral lymph nodes of C57BL/6 *WT, ISG15KO USP18C61A* mice. Cells were cultured for 36–48 h in RPMI with 2 μg ml^−1^ concanavalin A (Sigma) and subsequently 50 U ml^−1^ human recombinant IL-2 (Glaxo) was added to the medium every 2 days for at least 4–6 days, to obtain differentiated T lymphoblasts.

### Exosome purification

Cells were cultured in medium supplemented with 10% exosome-depleted FBS (bovine exosomes were removed by overnight centrifugation at 100,000 *g*). Supernatant fractions were collected from 16 to 20 h cell culture supernatants and exosomes were obtained by serial centrifugation as follows. Cells were pelleted (320 × *g* for 10 min) and the supernatant was centrifuged at 2,000 *g* for 15 min, to discard debris and dead cells. The supernatant was collected and ultracentrifuged at 10,000 *g* for 30 min at 4 °C (Beckman Coulter Optima L-100 XP, Beckman Coulter) and exosomes were pelleted by ultracentrifugation at 100,000 *g* for 70 min at 4 °C. The exosome pellet was washed in PBS and collected by ultracentrifugation at 100,000 *g* for 70 min (Beckman Coulter Optima L-100 XP, Beckman Coulter).

### Nanoparticle tracking analysis

Exosome numbers and size distribution were determined by measuring the rate of Brownian motion using a NanoSight LM10 system, which is equipped with fast video capture and particle-tracking software (NanoSight, Amesbury, UK). Samples were diluted before analysis to between 2 × 10^8^ and 20 × 10^8^ particles per ml, and the relative concentration was calculated according to the dilution factor. Data analysis was performed with *NTA 2.*1 software (Nanosight). Samples were analysed using manual shutter and gain adjustments, which resulted in shutter speeds of 15 or 30 ms, with camera gains between 280 and 560. The detection threshold was kept above 2; blur: auto; minimum expected particle size: 50 nm.

### ISGylation induction by overexpression

HEK293 cells were co-transfected with plasmids encoding human E1, E2 and E3 ligases (kindly provided by A. García-Sastre, Mount Sinai Hospital, NYC) together with plasmids encoding WT or C-terminal mutated human ISG15 fused to the V5 tag. When indicated, cells were co-transfected with the appropriate small interfering RNAs or with the EGFP-Rab7A T22N plasmid (gift from Qing Zhong, Addgene plasmid 28048). Cells were transfected using Lipofectamine 2000 (Invitrogen). Twenty-four hours after transfection, cells were cultured in exosome-depleted medium for 20 h and exosomes were isolated from supernatants as described above. When indicated, cells were incubated overnight in exosome-depleted medium containing 20 nM Bafilomycin A1 (Merck).

### Exosome extraction from blood serum

WT, *ISG15KO* and *USP18C61A* mice were treated with 5 μg g^−1^ body weight poly (I:C) (InvivoGen) intraperitoneally. Blood samples were extracted from the heart 24 h after poly (I:C) injection and allowed to clot 3 h at room temperature. Blood samples were kept 2 h at 4 °C and serum was obtained by centrifugation at 400 *g*, 10 min at 4 °C. Cell debris were removed by centrifugation at 3,000 *g*, 15 min at 4 °C and serum samples were stored at −20 °C. Exosomes were extracted from serum as previously described^70^; briefly, 125 μl of Exoquick reagent were added to 250 μl of serum and incubated overnight at 4 °C. Samples were centrifuged at 1,500 *g*, 30 min and exosome pellets resuspended in RIPA buffer.

### Gene silencing

HEK293 cells were transfected using Lipofectamine 2000 (Invitrogen) with the following small interfering RNAs: control (scrambled), ATG5 and TSG101 (SIGMA). Twenty-four hours after transfection, cells were cultured in exosome-depleted medium for 20 h and exosomes were isolated from supernatants as described above.

### Western blotting

Cells or exosomes were lysed in RIPA buffer (50 mM Tris-HCl pH 8, 150 mM NaCl, 1% Triton X-100, 0.1% sodium deoxycholate and 0.1% SDS) containing a protease inhibitor cocktail (Complete, Roche). Proteins were separated on 10% acrylamide/bisacrylamide gels and transferred to a nitrocellulose membrane. Membranes were incubated with primary antibodies (1:1,000) and peroxidase-conjugated secondary antibodies (1:5,000), and proteins were visualized with LAS-3000. The following antibodies were used: rabbit anti-human ISG15 (Proteintech, 15981-1-AP), mouse anti-human CD63 (Calbiochem, OP171), mouse 5A6 anti-human CD81 (Santa Cruz, sc-23962), mouse anti-human TSG101 (Abcam, GTX70255), mouse anti-GFP (Living colors, 632381), rabbit anti-p62 (Sigma, P0067), mouse DM1A anti-tubulin (Sigma, F2168), rabbit anti-Calnexin (Abcam, ab10286), goat anti-mouse peroxidase (Thermo Scientific), goat anti-rabbit peroxidase (Thermo Scientific) and donkey anti-goat peroxidase (Thermo Scientific). Full immunoblots are provided in Supplementary Fig. 6.

### Fluorescence confocal microscopy

For immunofluorescence assays, cells were plated onto slides coated with fibronectin (20 μg ml^−1^), incubated for 16 h min, fixed with 2% paraformaldehyde and stained with the indicated primary antibodies (1:100) followed by secondary antibodies (1:500). When indicated, cells were previously transfected with GFP-Rab5CA (Q79L) plasmid (gift from Sergio Grinstein, Addgene plasmid 35140) or LC3-GFP-RFP tandem construct (gift from Tamotsu Yoshimori, Addgene plasmid 21074). Samples were examined with a Leica SP5 confocal microscope (Leica) fitted with a × 63 objective and images were processed and assembled using Leica software. The following antibodies were used: anti-CD63 (clone Tea 3/18, generated in the laboratory), anti-HRS (Abcam, ab72053), anti-EE1A (Santa Cruz, sc-6415), anti-LAMP1-647 (BioLegend, 328612), anti-p62 (Sigma, P0067), goat anti-mouse-488 and goat anti-rabbit-RX (Life Technologies). Images were processed and quantified using LAS-AF and ImageJ.

### Cloning

ISG15-GFP plasmids were generated by excision of ISG15 from the pCAGG plasmid with XhoI and EcoICRI restriction enzymes, followed by insertion between the XhoI and AfeI sites of pAcGFP-N1. ISG15 stop codons were mutated using the QuickChange Site-Directed Mutagenesis kit (Agilent). ISG15-TSG101-GFP was obtained by inserting TSG101 into ISG15-GFP plasmid with XhoI and BamHI restriction enzymes. Non-de-ISGylable mutant ISG15-TSG101-GFP-AA was obtained by mutated terminal glycines into alanines using the QuickChange Site-Directed Mutagenesis kit (Agilent). Ubiquitin-GFP expressing plasmid was obtained from Addgene (gift from Nico Dantuma, Addgene plasmid 11928).

### Protein aggregation

HEK293 cells were transfected with GFP or ISG15-GFP plasmids using Lipofectamine 2000 (Invitrogen) and 48 h later lysed in lysis buffer (10 mM Tris-HCl pH 8, 100 mM NaCl, 1 mM EDTA, 0.5% NP40 and 50 mM iodoacetamide) supplemented with protease inhibitor cocktail (Complete, Roche). Cells were centrifuged for 5 min a 13,000 *g*, and the supernatant (S1) and pellet (P1) were saved. P1 was resuspended in lysis buffer containing 2% SDS, sonicated and centrifuged at 16,000 *g* for 10 min. The pellet (P2) was resupended in lysis buffer containing 2% SDS and sonicated. S1 and P2 were loaded on a poly-acrylamide gel and blotted for GFP. Subcellular fractionation was performed with ProteoExctarct Subcellular Proteome Extraction kit (Calbiochem).

### Protein degradation

HEK293 cells were cultured in six-well-plates and transfected with GFP or ISG15-GFP using Lipofectamine 2000 (Invitrogen). Cells were treated with 40 μg ml^−1^ cycloheximide (Sigma) to inhibit protein synthesis and were lysed at the indicated time points. When indicated, the medium contained 20 nM Bafilomycin A1 (Merk) or 40 μM MG132 (Sigma).

### Immunoprecipitation

1 × 10^7^ HEK293 cells per condition were lysed (25 mM Tris pH 8, 150 mM NaCl, 2 mM MgCl_2_, 0.5% NP-40 and protease inhibitors) and incubated for pre-clearing with pre-washed Protein G Dynabeads (Invitrogen; 50 μl per condition; 1 h, 4 °C). Fifty microlitres of Dynabeads per condition were washed twice in 0.01% Tween PBS and resuspended in 200 μl of 0.01% Tween PBS containing 5 μg mouse anti-V5 antibody (Life Technologies) per condition and incubated 1 h at 4 °C. Pre-cleared lysates were incubated with antibody-conjugated Dynabeads (1.5 h, 4 °C). Antibody-conjugated Dynabeads were washed six times with lysis buffer and transferred to clean tubes. Protein loading buffer was added, samples were boiled at 95 °C for 5 min and processed for immunoblotting.

### Electron microscopy and MVB quantification

Cells cultured on dishes were washed in PBS and fixed for 1 h in 2.5% glutaraldehyde in 0.1 M phosphate buffer at room temperature. Then, cells were slowly and gently scrapped and pelleted in eppendorf tubes. Pellets were washed in phosphate buffer and incubated with 1% OsO_4_ for 90 min at 4 °C. Then, samples were dehydrated, embedded in Spurr and sectioned using Leica ultramicrotome (Leica Microsystems). Ultrathin sections (50–70 nm) were stained with 2% uranyl acetate for 10 min and with a lead-staining solution for 5 min and observed using a transmission electron microscope, JEOL JEM-1010 fitted with a Gatan Orius SC1000 (model 832) digital camera.

For the calibration of images, quantification and analysis ImageJ was used. MVBs were identified and counted by morphology, having only discrete ILVs. Lysosomes contain multilamellar profiles. At least 20 MVBs were analysed per experiment from separate cells. Data were analysed from duplicate or triplicate separate experiments and two to four grids were used for each condition. The minimum number of cells scored for each condition was 20. Box scatter plots were generated using Prism GraphPad software and statistical tests were performed in Microsoft Excel. Data are means±s.d. and ***P-value<0.0001.

### Data availability

The data that support the conclusions of this study are available from the corresponding author upon request.

## Additional information

**How to cite this article:** Villarroya-Beltri, C. *et al*. ISGylation controls exosome secretion by promoting lysosomal degradation of MVB proteins. *Nat. Commun.* **7,** 13588 doi: 10.1038/ncomms13588 (2016).

**Publisher's note**: Springer Nature remains neutral with regard to jurisdictional claims in published maps and institutional affiliations.

## Supplementary Material

Supplementary Information Supplementary figures 1-6.
