# Supplementary material for: ISGylation controls exosome secretion by promoting lysosomal degradation of MVB proteins
Source: Nat Commun. 2016 Nov 24;7:13588. doi: 10.1038/ncomms13588 (PMC5123068; doi:10.1038/ncomms13588)
Supplement: Supplementary Information — Supplementary figures 1-6. [file ncomms13588-s1.pdf]

SUPPLEMENTARY FIGURES

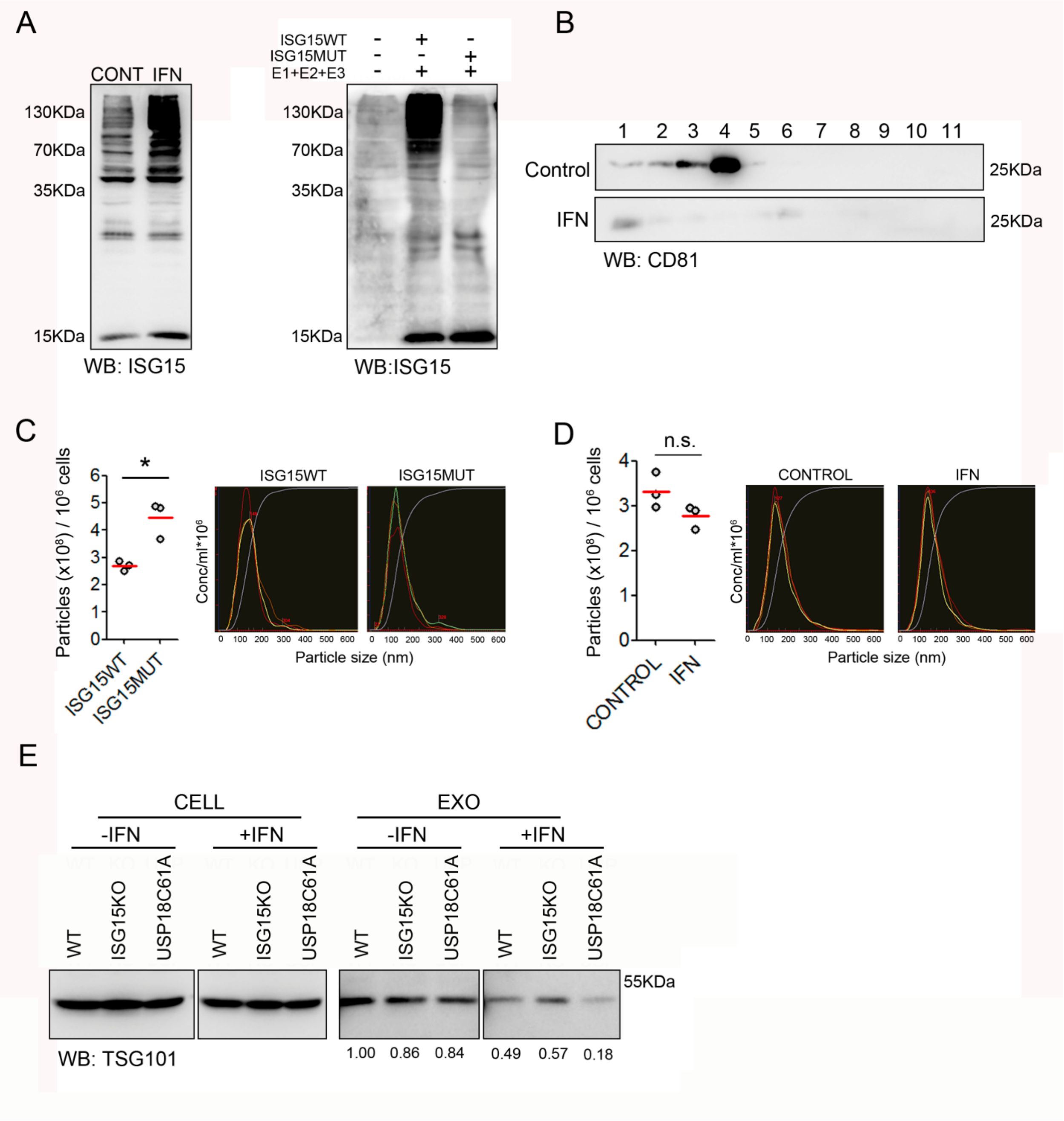

Supplementary Figure 1. (A) Left, western blot analysis of ISGylated proteins in Jurkat T cells treated with 1000U·ml^{-1} IFN for 16h (IFN) or left untreated (CONT); right, western blot analysis of ISGylated proteins in HEK293 cells non-transfected or co-transfected with plasmids encoding the ISGylation machinery and functional (ISG15WT) or mutated ISG15 (ISG15MUT). Cell lysates were blotted for ISG15. (B) Western blot analysis of the exosomal protein CD81 in sucrose fractions.

Extracellular Vesicles (EVs) obtained from equal numbers of Jurkat T cells treated 16h with IFN-I (IFN) or left untreated (Control) were laid on top of discontinuous sucrose gradient and ultracentrifugated overnight. Gradient fractions were collected and analyzed by immunoblot to show the distribution of exosomal CD81 in the sucrose fractions from lower (left) to higher sucrose densities (right). (C) Number of particles and size distribution analysis by Nanoparticle Tracking Analysis (NTA) of the EVs obtained from the supernatant of HEK293 cells co-transfected with human E1, E2 and E3 plasmids together with mutated (ISG15MUT) or wild type ISG15 plasmids (ISG15WT) and (D) control and IFN-treated Jurkat cells. Each dot shows the number of secreted particles purified from equal number of cells measured in three videos of 30s and mean is indicated in red lines. (E) Western blot analysis of the EVs obtained from equal numbers of *WT, ISG15KO* and *USP18C61A* T lymphoblasts treated 16h with IFN-I (IFN) or left untreated (CONT). Cells and EVs (EXO) were blotted for TSG101 and quantification of TSG101 protein levels in exosomal fractions respect to untreated WT condition is shown for a representative experiment.

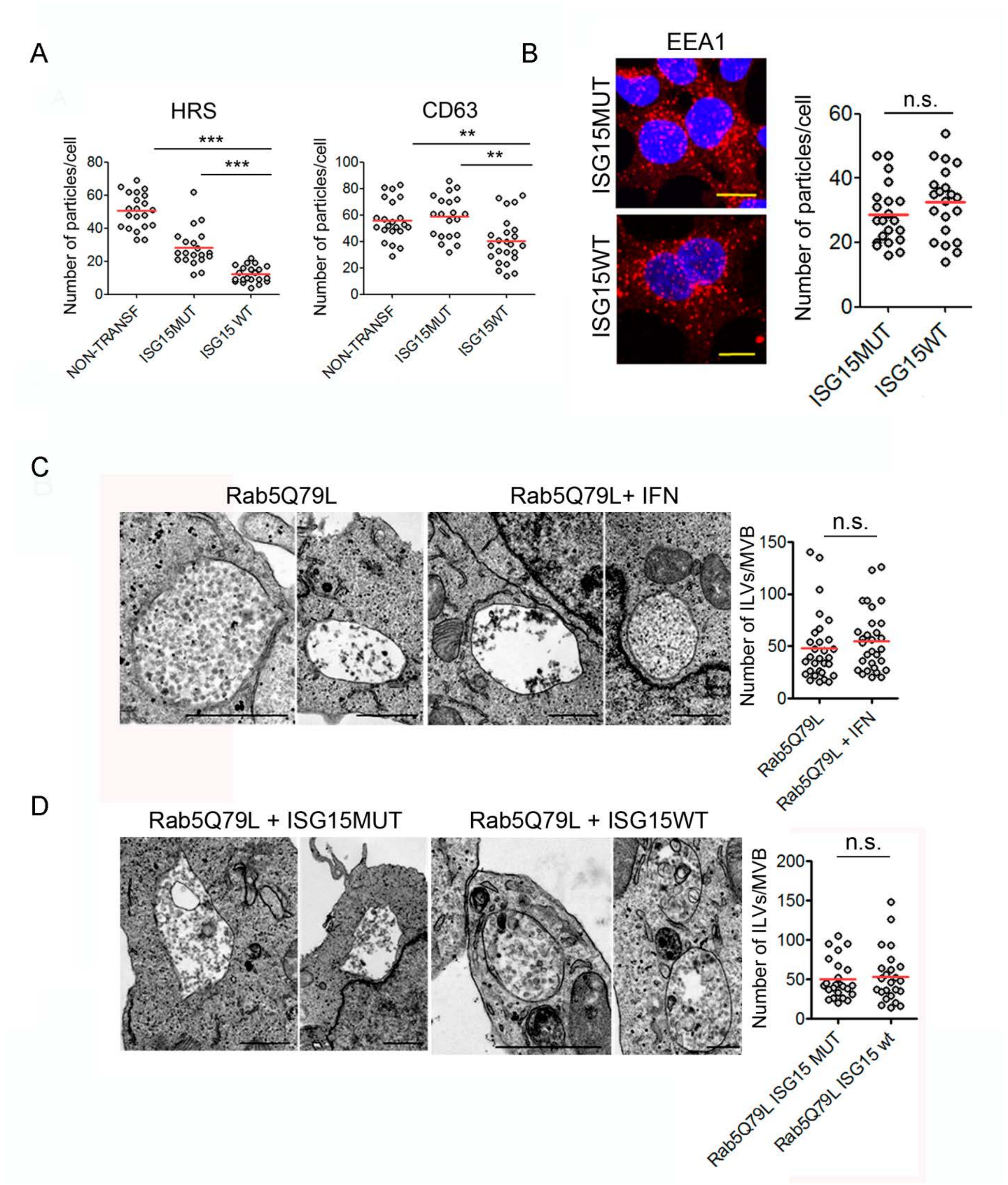

Supplementary Figure 2. (A) Quantification of HRS^{+} and CD63^{+} average number of particles per cell in confocal microscopy images in non-transfected HEK293 cells or HEK293 cells co-transfected with plasmids encoding the ISGylation machinery and the functional (ISG15WT) or mutated ISG15 (ISG15MUT) showed in Figure 2A. Images were processed by ImageJ, and the HRS^{+} and CD63^{+}

average number of particles per cell was quantified (n=21). Each dot represents the average particle size from individual cells and mean is indicated in red lines. (B) Confocal microscopy analysis of the early endosome marker EEA1 (red) in HEK293 cells co-transfected with human E1, E2 and E3 plasmids together with mutated (ISG15MUT) or wild type (ISG15WT) ISG15 plasmids. Nuclei were stained with DAPI. Images were processed by ImageJ, and number of EEA1^{+} particles was quantified (n=20). Each dot shows the average number of particles per cell and mean is indicated in red lines. (C) Electron microscopy images showing MVBs in HEK293 cells transfected with Rab5-Q79L-GFP mutant and treated with 1000U·ml^{-1} IFN-I, when indicated. Images were processed by ImageJ, and the number of ILVs per MVB was quantified (n=30). Each dot represents the number of ILVs per MVB and mean is indicated in red lines. Scale bar 1µm. (D) Electron microscopy images showing MVBs in HEK293 cells co-transfected with Rab5-Q79L-GFP mutant, the ISGylation machinery and functional (ISG15WT) or mutated ISG15 (ISG15MUT). Images were processed by ImageJ, and the number of ILVs per MVB was quantified (n=24). Each dot represents the number of number of ILVs per MVB and mean is indicated in red lines. Scale bar 1µm. t-test, n.s: p-value>0.05. **p-value<0.001, ***p-value<0.0001.

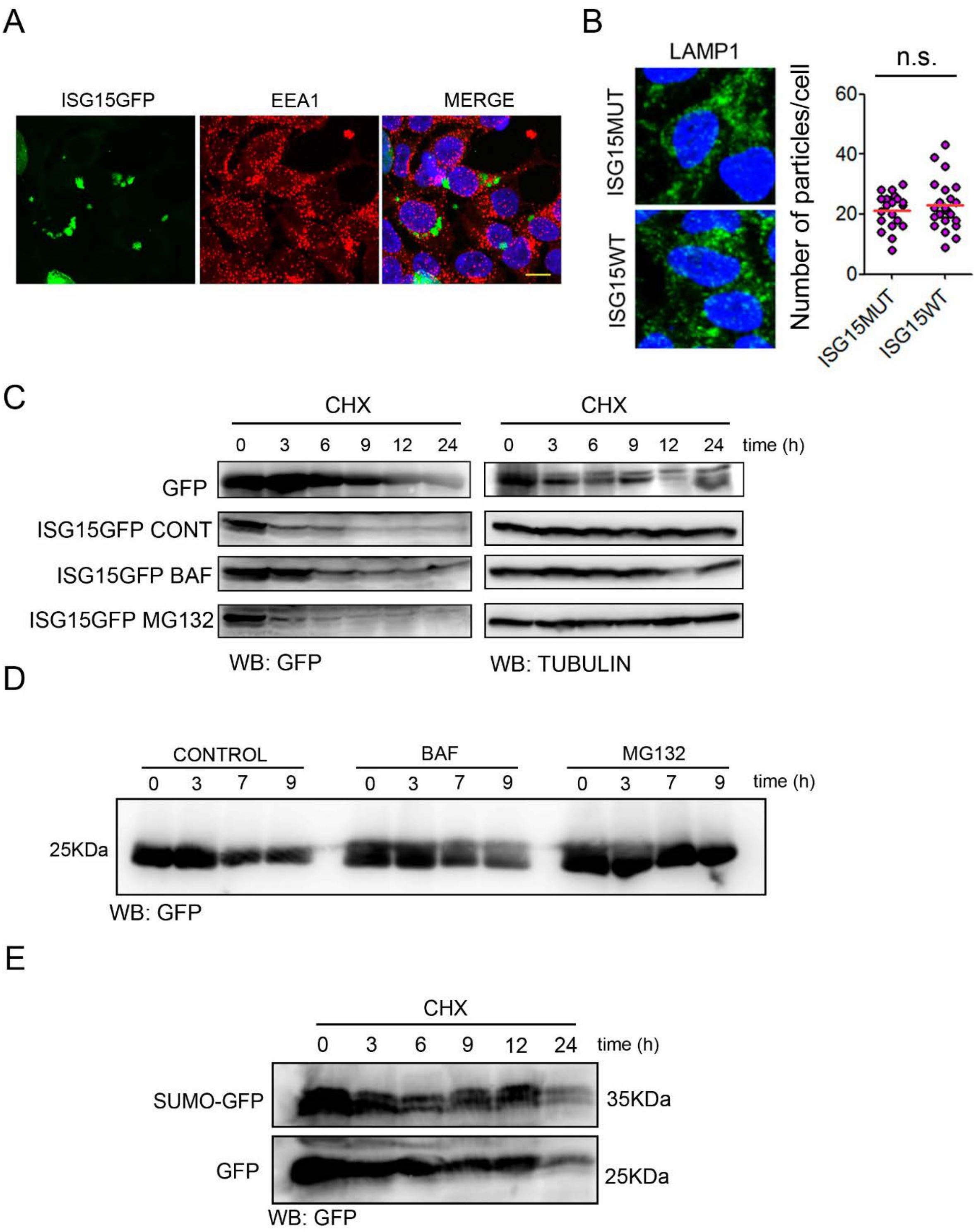

Supplementary Figure 3. (A) Confocal analysis of ISG15GFP (green) co-localization with the early endosome marker EE1A (red). Scale bar 10µm. Nuclei were stained with DAPI. (B) Confocal

microscopy analyses of the lysosome marker LAMP1 (green) in HEK293 cells co-transfected with human E1, E2 and E3 plasmids together with mutated (ISG15MUT) or wild type (ISG15WT) ISG15 plasmids. Nuclei were stained with DAPI. Images were processed by ImageJ, and number of LAMP1$^+$ particles was quantified (n=20). Each dot shows the average number of particles per cell and mean is indicated in red lines. (C) Western blot analysis of GFP and ISG15-GFP degradation kinetics in HEK293 cells transfected with GFP or ISG15-GFP and treated with cycloheximide to inhibit protein synthesis during the indicated times. Where specified, the medium was supplemented with the lysosome inhibitor Bafilomycin A1 (BAF) or the proteasome inhibitor MG132. Cell lysates were blotted against GFP and Tubulin was used as loading control. (D) Western blot analysis of GFP degradation kinetics in HEK293 cells transfected with GFP and treated with cycloheximide to inhibit protein synthesis during the indicated times. Where specified, the medium was supplemented with the lysosome inhibitor Bafilomycin A1 (BAF) or the proteasome inhibitor MG132. Cell lysates were blotted against GFP. (E) Western blot analysis of SUMO-GFP degradation kinetics in HEK293 cells transfected with SUMO-GFP or GFP and treated with cycloheximide to inhibit protein synthesis during the indicated times. Cell lysates were blotted against GFP.

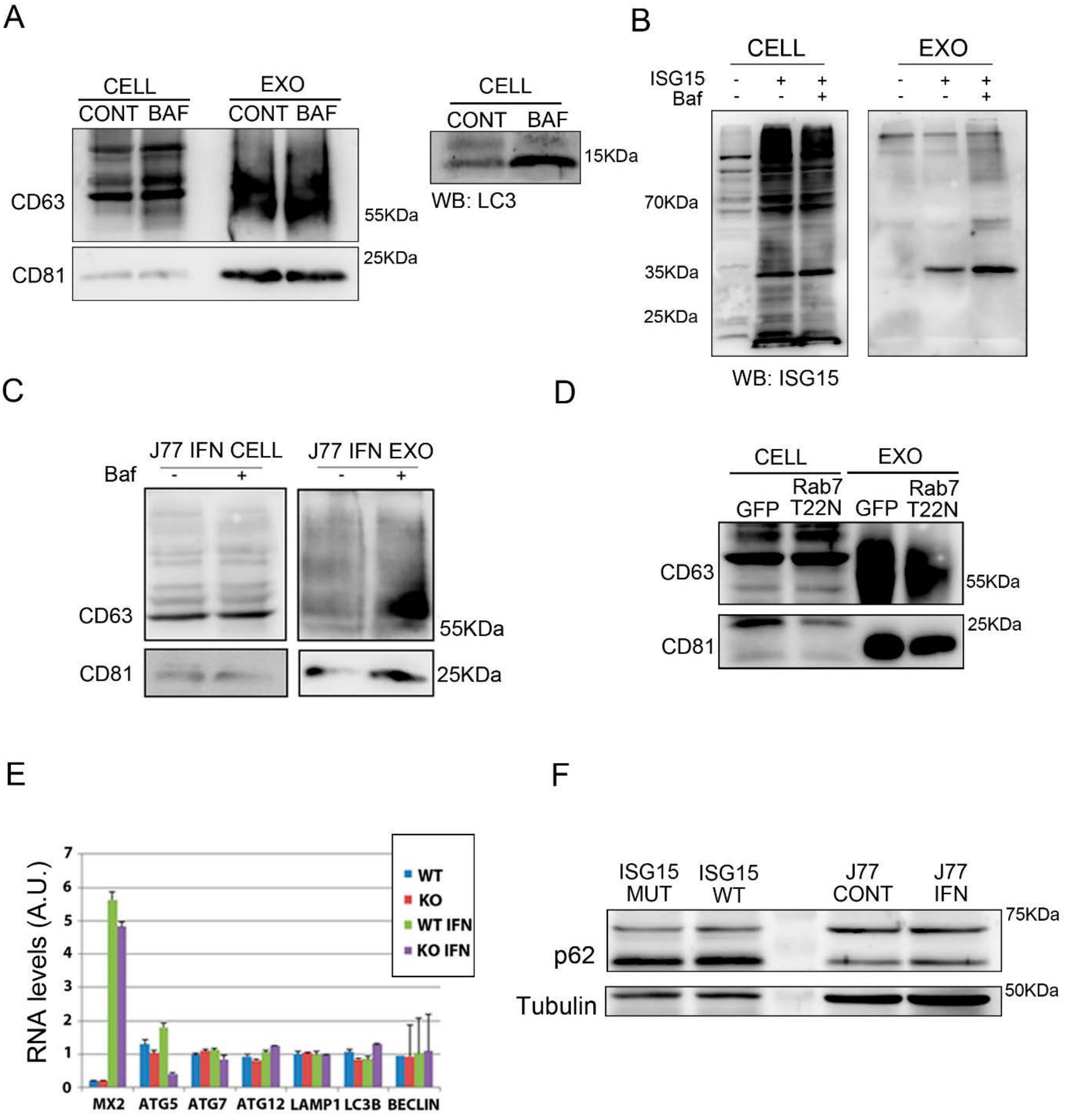

Supplementary Figure 4. (A) Western blot analysis of exosome secretion in HEK293 cells left untreated (CONT) or treated with Bafilomycin A1 (BAF). Cells and EVs (EXO) were blotted for CD63 and CD81. Right, cell lysates were blotted for LC3 to verify the proper function of Bafilomycin A1. (B) Western blot analysis of cells and EVs protein ISGylation in non-transfected HEK293 cells and HEK293 cells co-transfected with plasmids encoding the ISGylation machinery, ISG15 and treated with Bafilomycin A1 when indicated. Cells and EVs (EXO) were blotted for ISG15. (C)

Western blot analysis of EVs purified from equal numbers of Jurkat T cells treated 16h with 1000U·ml^{-1} IFN-I and Bafilomycin A1, when indicated. Cells and EVs (EXO) were blotted for CD63 and CD81. (D) Western blot analysis of exosome secretion in HEK293 cells transfected with GFP or the Rab7 dominant negative mutant Rab7T22N. Cells and EVs were blotted for CD63 and CD81. (E) qPCR analyses of mRNA levels of IFN-I-activated genes (MX2) or autophagy genes in wild-type (WT) or ISG15KO (KO) primary T lymphoblasts treated with IFN-I or left untreated. (F) Western blot analyses of p62 levels in HEK 293T cells co-transfected with human E1, E2 and E3 plasmids together with mutated ISG15 (ISG15MUT) or wild type (ISG15WT) plasmids, or Jurkat T cells untreated (CONT) or treated with IFN-I (IFN).

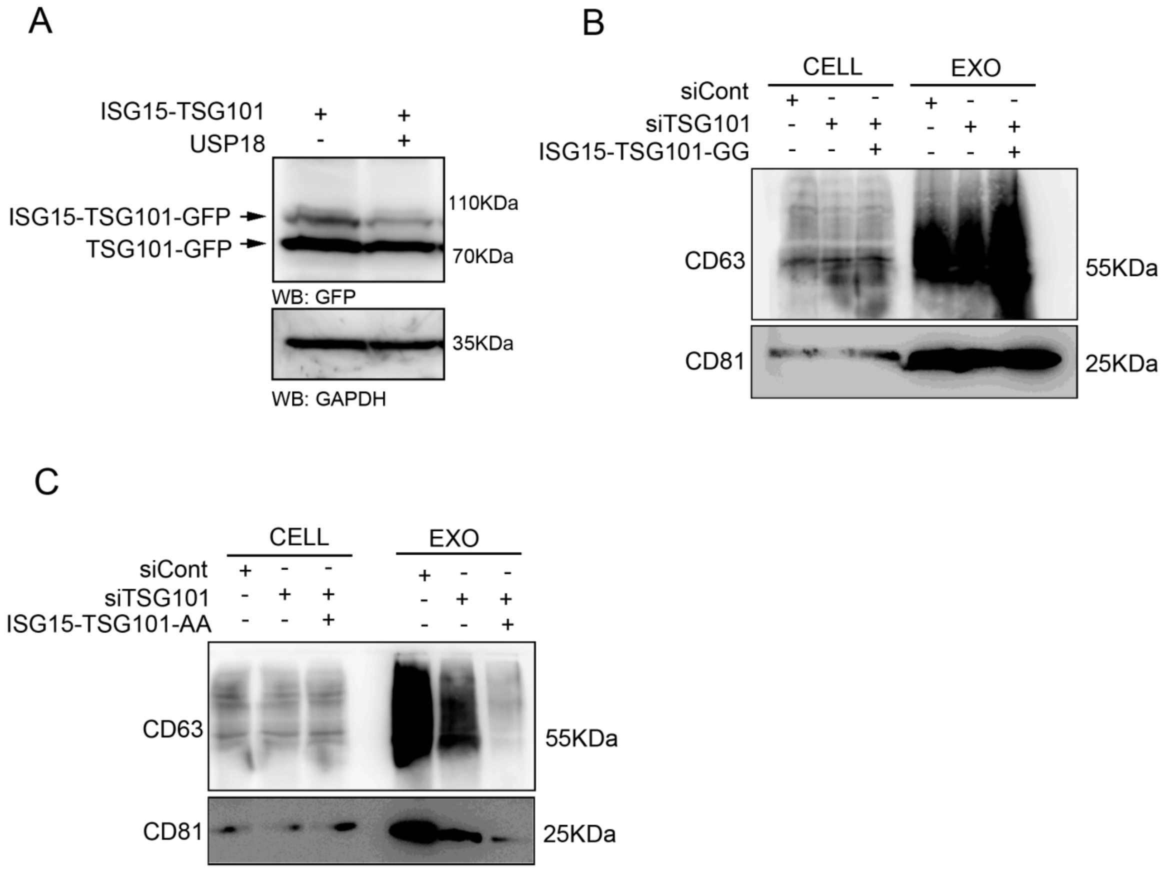

Supplementary Figure 5. (A) Western blot analysis of TSG101 ISGylation in HEK293 cells transfected with ISG15-TSG101-GFP and USP18 when indicated. Cell lysates were blotted for GFP and GAPDH was used as loading control. Arrows indicate the expected molecular wights of ISGylated (ISG15-TSG101-GFP) and non-ISGylated TSG101-GFP. (B) Higher exposure of the western blots shown in Figure 6F from HEK293 cells transfected with control siRNAs or siRNAs targeting TSG101. When indicated, cells were co-transfected with ISG15-TSG101-GFP-GG. Cells and EVs (EXO) were blotted for CD63 and CD81. (C) Higher exposure of the western blots shown in Figure 6G from HEK293 cells transfected with control siRNAs or siRNAs targeting TSG101. When indicated, cells were co-transfected with the non-de-ISGylable mutant ISG15-TSG101-GFP-AA. Cells and EVs (EXO) were blotted for CD63 and CD81.

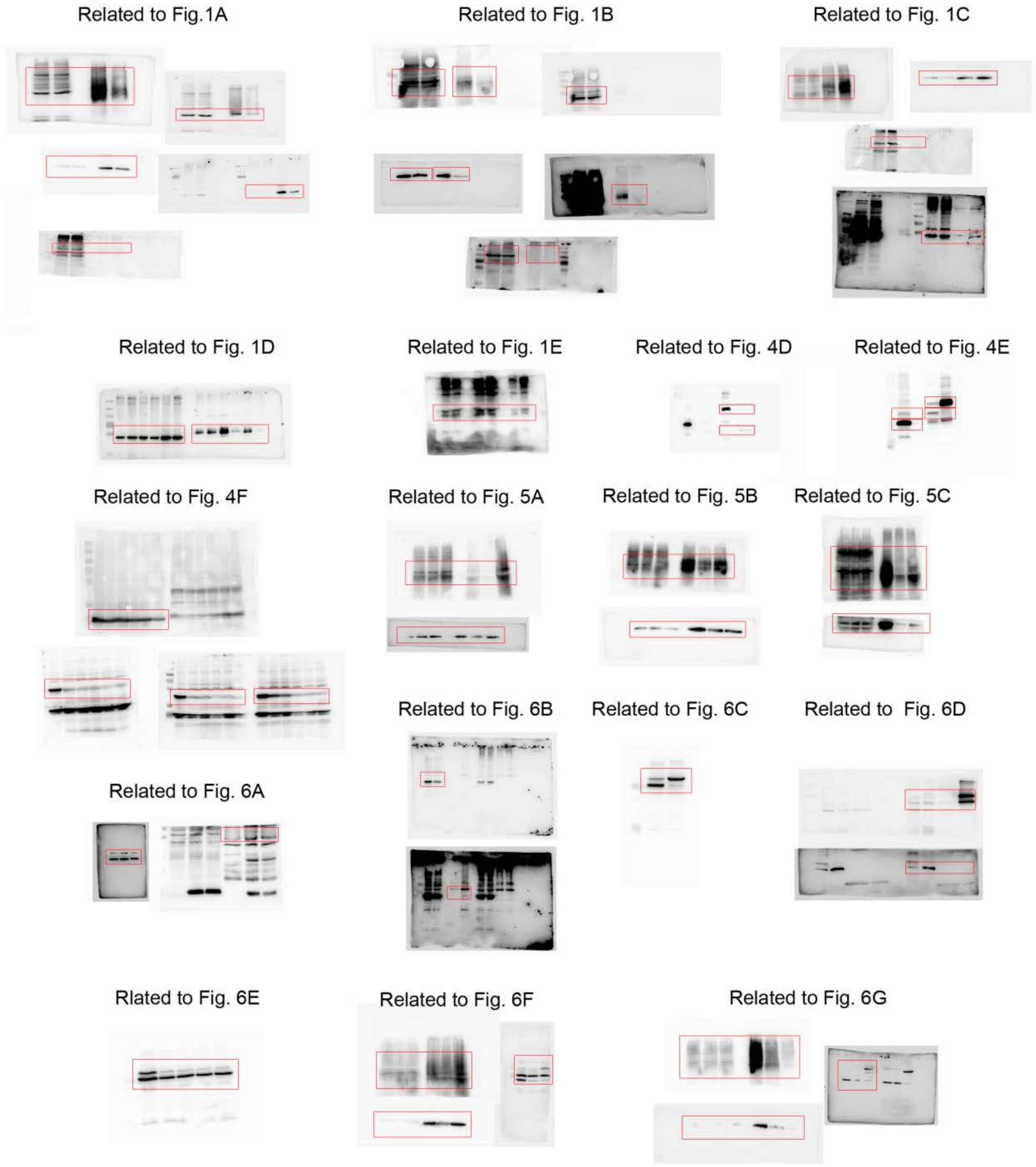

Supplementary Figure 6. Full immunoblots. Full immunoblots with indicated areas of selection.
